# Cardiovascular disease prevention knowledge and associated factors among adults in Mukono and Buikwe districts in Uganda

**DOI:** 10.1186/s12889-020-09264-6

**Published:** 2020-07-22

**Authors:** Rawlance Ndejjo, Fred Nuwaha, Hilde Bastiaens, Rhoda K. Wanyenze, Geofrey Musinguzi

**Affiliations:** 1 Department of Disease Control and Environmental Health, School of Public Health, College of Health Sciences, Makerere University, Kampala, Uganda; 2 Department of Primary and Interdisciplinary care, Faculty of Medicine and Health Sciences, University of Antwerp, Antwerp, Belgium

**Keywords:** Diet, Healthy lifestyles, Hypertension, Physical activity and sub-Saharan Africa

## Abstract

**Background:**

With the growing epidemic of Cardiovascular Disease (CVD) in sub-Saharan Africa, behavioural change interventions are critical in supporting populations to achieve better cardiovascular health. Population knowledge regarding CVD is an important first step for any such interventions. This study examined CVD prevention knowledge and associated factors among adults in Mukono and Buikwe districts in Uganda.

**Methods:**

The study was cross-sectional in design conducted among adults aged 25 to 70 years as part of the baseline assessment by the Scaling-up Packages of Interventions for Cardiovascular disease prevention in selected sites in Europe and Sub-Saharan Africa (SPICES) – project. Data were collected using pretested semi-structured questionnaires, and respondents categorized as knowledgeable if they scored at least five out of six in the knowledge questions. Data were exported into STATA version 15.0 statistical software for analysis conducted using mixed-effects Poisson regression with fixed and random effects and robust standard errors.

**Results:**

Among the 4372 study respondents, only 776 (17.7%) were knowledgeable on CVD prevention. Most respondents were knowledgeable about foods high in calories 2981 (68.2%), 2892 (66.1%) low fruit and vegetable intake and high salt consumption 2752 (62.9%) as CVD risk factors. However, majority 3325 (76.1%) thought the recommended weekly moderate physical activity was 30 min and half 2262 (51.7%) disagreed or did not know that it was possible to have hypertension without any symptoms. Factors associated with high CVD knowledge were: post-primary education [APR = 1.55 (95% CI: 1.18–2.02), *p* = 0.002], formal employment [APR = 1.69 (95% CI: 1.40–2.06), *p* < 0.001] and high socio-economic index [APR = 1.35 (95% CI: 1.09–1.67), *p* = 0.004]. Other factors were: household ownership of a mobile phone [APR = 1.35 (95% CI: 1.07–1.70), *p* = 0.012] and ever receiving advice on healthy lifestyles [APR = 1.38 (95% CI: 1.15–1.67), *p* = 0.001].

**Conclusions:**

This study found very low CVD knowledge with major gaps around recommended physical activity duration, diet and whether hypertension is asymptomatic. Observed knowledge gaps should inform suitable interventions and strategies to equip and empower communities with sufficient information for CVD prevention.

**Trial registration:**

ISRCTN Registry ISRCTN15848572, January 2019, retrospectively registered.

## Background

In sub-Saharan Africa (SSA), many countries are facing an epidemic of non-communicable diseases contributing to morbidity, mortality, and loss of productivity and economic growth [1–3]. Among the non-communicable conditions, cardiovascular disease (CVD) is a leading cause of death and disability contributing over 1 million deaths in 2013, 5.5% of all global deaths that year [1]. The growing burden of CVD in SSA, expected to double by 2030, is attributed to population growth, aging and changing lifestyles regarding physical activity, diets, alcohol consumption and tobacco use [1, 3]. In Uganda, the prevalence of physiological risk factors such as hypertension [4] and other determinants including alcohol consumption, unhealthy diets and overweight are high and rising [4, 5]. In Mukono and Buikwe districts, among persons aged above 15 years and above, 21.8% have high blood pressure but with low levels of status awareness and control [6], much like the rest of the country [4].

To deal with the rising burden of CVD in SSA, innovative behavioural change interventions are warranted to address the rising risk factors and support populations to achieve better cardiovascular health outcomes. Population knowledge regarding CVD is an important precursor for any such interventions [7]. Indeed, a knowledgeable population is more likely to make healthier lifestyle choices, recognize disease risk factors and adopt positive health seeking behaviours [8]. Unfortunately, CVD is a relatively new phenomenon in SSA, and thus most of the population has a limited understanding of the disease and related risk factors. Moreover, available data is inadequate with only a handful of studies examining knowledge [9, 10] and usually with a focus on specific conditions such as stroke [11, 12] and risk factors such as hypertension [13]. To generate evidence for targeted interventions, this study examined knowledge on CVD prevention and associated factors among adults in Mukono and Buikwe districts in Uganda.

## Methods

### Study design and area

This cross-sectional study was informed by data from the baseline survey of a stepped wedge trial of the Scaling-up Packages of Interventions for Cardiovascular disease prevention in selected sites in Europe and Sub-Saharan Africa (SPICES) – project [14]. In Uganda, the project is being implemented in Mukono and Buikwe districts located 35.0 and 57.4 km from Uganda’s capital, Kampala, with a total population of 1,000,000, of whom 50.0% are female [15]. Residents in these districts, 70% of whom live in rural areas, majorly practice subsistence farming, fishing and a section engages in small scale trade.

### Study population, sample size and sampling

The study population were residents in the two districts aged between 25 and 70 years with households as study units. The sample size was calculated following Hemming’s formula for a stepped wedge trial as previously described [14]. In the districts, 20 parishes, each that hosted a Health Centre III, were selected. The HC III is situated at the sub-county level with a general outpatient clinic and a maternity ward receiving referrals from HC II (at parish) and HC I (at village) levels and refers to higher levels (HC IV and general hospital) within the district. From each parish, a random sample of four villages was drawn to form the study clusters. A mapping of area boundaries and listing of all households was conducted prior to the study to generate a sampling frame [14]. In the selected villages, a random sample of 200 households was obtained and all adults aged 25–70 years within the households interviewed. Analysis for this paper is based on 4372 respondents from 3689 randomly selected households across 80 villages in the 20 parishes.

### Data collection

Data collection for this study was conducted between December 2018 and January 2019 by a team of trained Research Assistants in the study sites using pretested semi-structured questionnaires informed by the healthy-lifestyle counselling module of the WHO Hearts package that details the major behavioural risk factors for CVD [16]. The study developed and utilized two sets of questionnaires, the household and participant questionnaires. The household questionnaire (Additional file 1) captured household information including source of drinking water, type of toilet facility, cooking fuel, floor and roof material of house, household assets such as radio, television, mobile phone and vehicle, which supported development of the socio-economic index, and household income. On the other hand, the participant questionnaire (Additional file 2) had questions on socio-demographic characteristics of respondents including sex, age, education level and occupation, and knowledge on CVD prevention and lifestyle factors such as diet, physical activity, smoking and alcohol use [14]. The questionnaire also asked whether respondents had ever received advice on health lifestyles and who provided them this advice. The questionnaires were developed in English and translated into *Luganda*, the most spoken local language in the study area, and uploaded on Research Electronic Data Capture (REDCap) software designed with data entry checks and inbuilt skip patterns and installed on smart phones and tablets. Research Assistants approached respondents in their households to respond to questionnaires with household information collected first. Field work was overseen by a team of supervisors.

### Data management and analysis

All collected data were transmitted to the server located at the university and data downloaded, reviewed and cleaned and any discrepancies addressed. Data were then imported into STATA version 15.0 (Stata Corp, Texas, USA) statistical software for analysis. Data from both the household and participant questionnaires were merged using prior assigned household numbers. To obtain the composite knowledge score, responses to questions on knowledge were graded and assigned 1 if answered correctly and 0 if otherwise. A total score with the maximum as six was then generated for each respondent. The Cronbach’s alpha for the reliability of our knowledge scale was 0.6571. Respondents with a total score of five and above were classified as knowledgeable about CVD prevention (based on Bloom’s cut off point) and assigned a code 1 and the rest assigned a code of 0 to form a binary outcome variable for further data analysis. To obtain the socio-economic index, data collected on household characteristics and assets was fully dichotomized and principal component analysis used to obtain the first principal component explaining household wealth. This variable was then divided into quintiles and categorized into lowest 40% (low), middle 40% (middle) and upper 20% (high).

For the analysis, descriptive statistics were run to characterize the sample and proportions and means reported. Because the study outcome was not rare (> 10%) and the need to control for clustering at parish, village and household levels, prevalence ratios (PRs) were used as a measure of association [17, 18]. The PRs were obtained using mixed-effects Poisson regression analysis with fixed (individual characteristics) and random (parish, village, household) effects and robust standard errors. Separate models were run to obtain factors associated with CVD prevention knowledge at the bivariate level and thereafter, variables with a *p*-value of < 0.2 were considered for multivariate analysis. Multi-collinearity among independent variables was assessed using the Pearson correlation co-efficient and for any pair of variables with a co-efficient of > 0.4 and p-value < 0.05, only one was included in the multivariate model. Reporting for this study followed the STROBE checklist (Additional file 3).

## Results

### Socio-demographic characteristics of respondents

Of the 5314 eligible adults, 4372 (82.3%) responded to the survey among whom majority 2632 (60.2%) were female, 1761 (40.3%) were aged between 25 to 35 years (mean age [SD] = 41.4 (±12.7) and just over half 2284 (52.2%) had attained primary education. Most 3287 (76.0%) respondents were farmers or casual labourers, about four in ten 1858 (42.5%) had lived in their areas for over 25 years and just over a third 1357 (36.5%) of their households earned over $28 per month (Table 1).

**Table 1:** Socio-demographic characteristics of respondents

Characteristic	Total n (%)
Overall	4372 (100)
Sex	
Female	2632 (60.2)
Male	1740 (39.8)
Age in years (mean ± SD)	41.4 (±12.7)
25–35	1761 (40.3)
36–50	1572 (35.9)
Above 50	1039 (23.8)
Education level	
None	650 (14.9)
Primary	2284 (52.2)
Post-primary	1438 (32.9)
Occupation (*n* = 4325)	
Farming / casual labourer	3287 (76.0)
Business / informal small-scale trading	845 (19.5)
Formal employment such as civil servant	193 (4.5)
Monthly household income in Uganda shillings (USD) (*n* = 3718)	
< 50,000 ($14)	1060 (28.5)
50,000–100,000 ($14 - $28)	1301 (35.0)
> 100,000 ($28)	1357 (36.5)
Socio-economic index^a^ (*n* = 4253)	
Low	1702 (40.0)
Middle	1701 (40.0)
High	850 (20.0)
Religion (*n* = 4359)	
Catholic	1536 (35.2)
Protestant	1288 (29.5)
Muslim	843 (19.3)
Others (Pentecostal, traditionalists and no religion)	692 (15.9)
Marital status	
Never married	353 (8.1)
Cohabiting or married	2794 (63.9)
Divorced or widowed	1225 (28.0)
Respondent is household head	
No	1577 (36.1)
Yes	2795 (63.9)
Household had radio or television (*n* = 4253)	
No	1098 (25.8)
Yes	3155 (74.2)
Household had a mobile phone (*n* = 4253)	
No	687 (16.1)
Yes	3566 (83.9)
Duration in area	
Less than 5 years	910 (20.8)
5 to 5 years	1604 (36.7)
Above 25 years	1858 (42.5)
Where lived most adult life	
Rural	3711 (84.9)
Urban / city	661 (15.2)
Where lived most childhood life	
Rural	3932 (89.9)
Urban / city	440 (10.1)

^a^*Low = lowest 40%, middle = middle 40% and high = upper 20% of household wealth*

### Knowledge about cardiovascular disease prevention

Table 2 shows a summary of the respondents’ scores on CVD prevention knowledge questions. The mean score of respondents on the scale of 6 questions was 3.1 (±1.43) with most 3596 (82.2%) scoring below 5. Respondents were more knowledgeable about foods high in calories 2981 (68.2%), low fruit and vegetable intake 2892 (66.1%) and high salt consumption 2752 (62.9%) as risk factors for CVD. On the other hand, over three quarters of respondents 3325 (76.1%) thought the recommended weekly moderate physical activity was 30 min and a half 2262 (51.7%) disagreed or did not know that it was possible to have hypertension without any symptoms. Overall, 776 (17.7%) of respondents had high knowledge on CVD prevention.

**Table 2:** Knowledge on cardiovascular disease prevention

Knowledge statement (***n*** = 4372)	Yes (%)	No (%)	Don’t know (%)
Someone can have hypertension without any symptoms	2110 (48.3)	902 (20.6)	1360 (31.1)
High salt consumption can increase your risk of developing hypertension	2752 (62.9)	414 (9.5)	1206 (27.6)
Foods high in calories such as chips, sugary drinks, cakes are good for the heart.	444 (10.2)	2981 (68.2)	947 (21.7)
Increased consumption of fruits and vegetables increases your risk of cardiovascular disease.	445 (10.2)	2892 (66.1)	1035 (23.7)
Sitting for a long time puts you at risk for high blood pressure	2719 (62.2)	487 (11.1)	1166 (26.7)
In order to reduce your risk of cardiovascular disease, moderate physical activity of at least 30 min is recommended every week.	3325 (76.1)	155 (3.6)	892 (20.4)
**Overall knowledge [mean score (SD)]**	3.1 (±1.43)		
Low (score less than 5)	3596 (82.2)		
High (score 5 and above)	776 (17.7)		

### Advice on healthy lifestyles

**Fig. 1:**
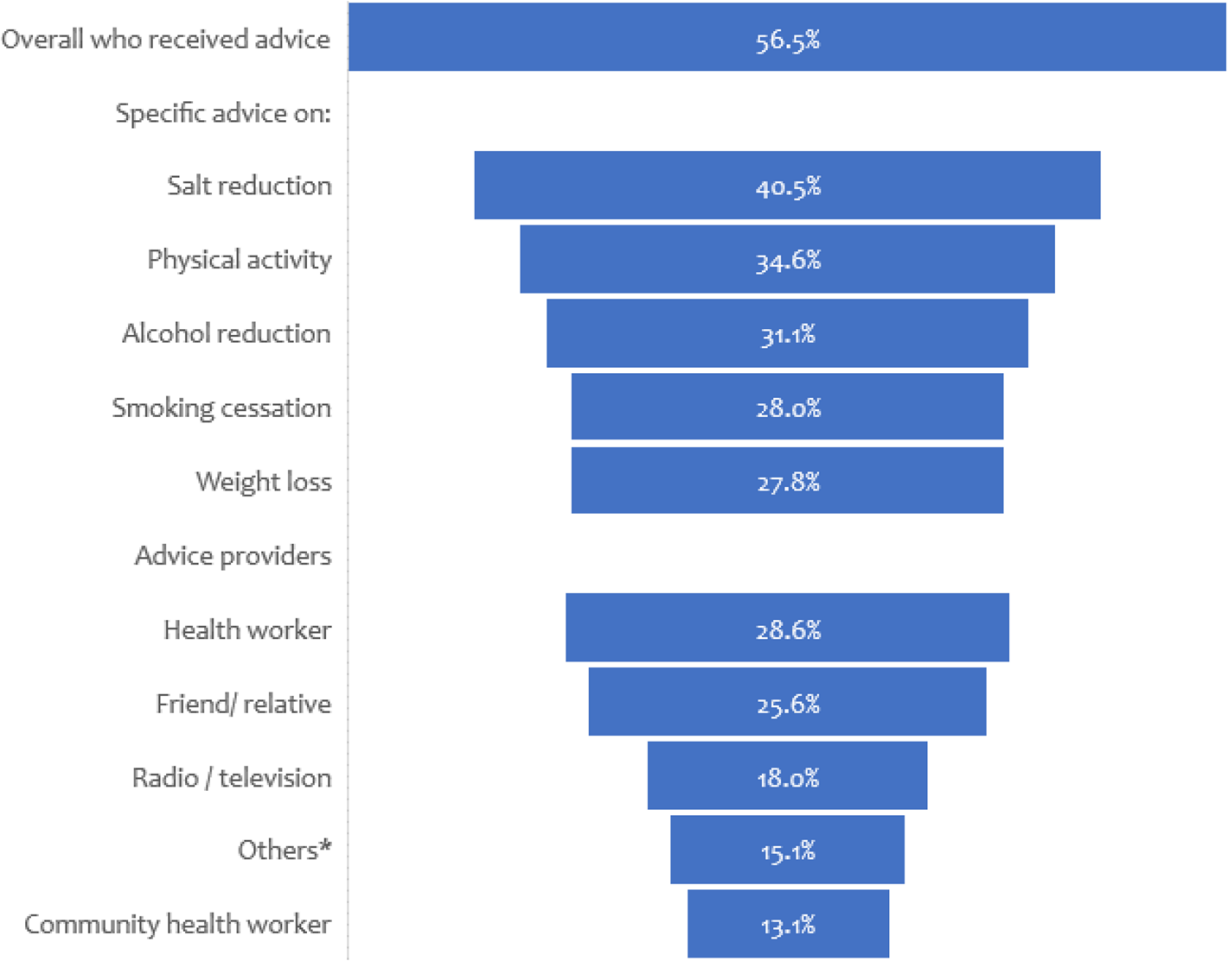
Advice on healthy lifestyles to community members. *Church, gym, herbalists, school, internet

Overall, over half of the respondents 2471 (56.5%) had received advice on at least one of the CVD risk factors with most reporting advice on salt reduction 1772 (40.5%) and physical activity 1512 (34.6%). The major providers of advice were health workers 1217 (28.6%) and friends / relatives 1090 (25.6%) (Fig. 1).

### Factors associated with knowledge on cardiovascular disease prevention

Bivariate analysis revealed that education level, occupation, household income, socio-economic index, religion and where respondent lived most adult or childhood life as significantly associated with knowledge on CVD prevention. Furthermore, households that had a radio or television, or a mobile phone and respondents who had ever received advice on healthy lifestyles had significantly higher knowledge. Due to multi-collinearity, place where most childhood was spent, whether respondent was household head and whether respondents had ever had their blood pressure measured were not included in the multivariate analysis. When qualifying variables where adjusted for in a multivariable model, knowledge on CVD prevention was significantly higher among respondents who had completed post-primary education [Adjusted PR = 1.55 (95% CI: 1.18–2.02), *p* = 0.002], those who were in formal employment [APR = 1.69 (95% CI: 1.40–2.06), *p* < 0.001] and those who belonged to the high socio-economic index [APR = 1.35 (95% CI: 1.09–1.67), *p* = 0.004]. Additionally, the proportion of respondents with high CVD prevention knowledge was 35 and 37% higher among those whose households had a mobile phone [APR = 1.35 (95% CI: 1.07–1.70), *p* = 0.012] and those who had ever received advice on healthy lifestyles [APR = 1.37 (95% CI: 1.14–1.65), *p* = 0.001] respectively (Table 3). There was no interaction between age and sex.

**Table 3:** Factors associated with knowledge on cardiovascular disease prevention

Characteristic	Knowledgeable (%)	PR [95% CI]	***p***-value	APR [95% CI]	***p***-value
Overall	754 (17.7)				
Sex					
Female	461 (17.5)	1		1	
Male	315 (18.1)	1.11 (0.99–1.23)	0.070	1.09 (0.96–1.25)	0.189
Age (years)					
25–35	344 (19.5)	1		1	
36–50	254 (16.2)	0.88 (0.72–1.06)	0.181	0.92 (0.76–1.12)	0.426
Above 50	178 (17.1)	0.95 (0.76–1.19)	0.674	1.12 (0.91–1.37)	0.290
Education level					
None	66 (10.1)	1		1	
Primary	335 (14.7)	1.37 (1.09–1.71)	**0.006**	1.18 (0.93–1.49)	0.176
Post-primary	375 (26.1)	2.15 (1.66–2.80)	**< 0.001**	1.55 (1.18–2.02)	**0.002**
Occupation (*n* = 4207)					
Farmer / casual labourer	498 (15.1)	1		1	
Business / informal small-scale trading	192 (22.7)	1.33 (1.13–1.58)	**0.001**	1.13 (0.93–1.37)	0.210
Formal employment such as civil servant	83 (43.0)	2.40 (2.01–2.87)	**< 0.001**	1.69 (1.40–2.06)	**< 0.001**
Household income (*n* = 3718)					
Less than 50,000	164 (15.5)	1		1	
50,000 - 100,000	230 (17.7)	1.18 (1.02–1.37)	**0.023**	1.00 (0.85–1.18)	0.966
Above 100,000	293 (21.6)	1.50 (1.23–1.84)	**< 0.001**	1.08 (0.90–1.30)	0.383
Socio-economic index^a^ (*n* = 4253)					
Low	210 (12.3)	1		1	
Middle	283 (16.6)	1.27 (1.03–1.58)	**0.028**	1.05 (0.83–1.32)	0.677
High	261 (30.7)	1.95 (1.52–2.49)	**< 0.001**	1.35 (1.09–1.67)	**0.004**
Religion (*n* = 4240)					
Catholic	244 (15.9)	1			
Protestant	242 (18.8)	1.15 (1.03–1.28)	**0.012**		
Muslims	149 (17.7)	1.08 (0.92–1.27)	0.370		
Others (traditionalists and no religion)	141 (20.4)	1.18 (0.93–1.49)	0.162		
Marital status					
Never married	56 (15.9)	1		1	
Cohabiting or married	515 (18.4)	1.15 (0.92–1.45)	0.217	1.15 (0.94–1.42)	0.174
Divorced or widowed	205 (16.7)	1.03 (0.78–1.36)	0.823	1.23 (0.97–1.57)	0.080
Respondent is household head					
No	268 (17.0)	1			
Yes	508 (18.2)	1.09 (0.92–1.29)	0.297		
Household had radio or television (*n* = 4253)					
No	154 (14.0)	1		1	
Yes	600 (19.0)	1.26 (1.04–1.52)	**0.019**	1.08 (0.88–1.34)	0.445
Household had a mobile phone (*n* = 4253)					
No	70 (10.2)				
Yes	684 (19.2)	1.63 (1.29–2.05)	**< 0.001**	1.35 (1.07–1.70)	**0.012**
Duration in area					
Less than 5 years	171 (18.8)	1			
5 to 25 years	294 (18.3)	1.01 (0.83–1.22)	0.933		
Above 25 years	311 (16.7)	0.99 (0.76–1.27)	0.916		
Where lived most adult life					
Rural	586 (15.8)	1			
Urban / city	190 (28.7)	1.35 (1.07–1.69)	**0.011**		
Where lived most childhood life					
Rural	667 (17.0)	1			
Urban / city	109 (24.8)	1.20 (1.02–1.42)	**0.028**		
Ever been told by health worker that had high blood pressure					
Yes	668 (17.4)	1		1	
No	108 (20.6)	1.17 (0.97–1.41)	0.096	1.05 (0.89–1.23)	0.546
Ever had blood pressure measured					
No	425 (16.4)	1			
Yes	351 (19.7)	1.12 (0.92–1.36)	0.266		
Ever received advice on healthy lifestyles					
No	248 (13.0)	1		1	
Yes	528 (21.4)	1.48 (1.22–1.78)	**< 0.001**	1.37 (1.14–1.65)	**0.001**

^a^*Low = lowest 40%, middle = middle 40% and high = upper 20% of household wealth*

## Discussion

This study examined knowledge on CVD prevention and associated factors among adults aged 25 to 70 years in Mukono and Buikwe districts in Uganda. Overall, knowledge levels were unsatisfactory with less than a fifth of respondents knowledgeable. Factors that were significantly associated with high CVD prevention knowledge were: post-primary education, formal employment, and high socio-economic index. In addition, respondents from households that had a mobile phone and those who had ever received advice on healthy lifestyles were significantly more knowledgeable about CVD prevention.

In our study, only 17.7% of the respondents had high knowledge on CVD prevention. This is consistent with findings from a systematic review on CVD knowledge and awareness among the general population in SSA [19]. Other studies that examined knowledge on specific CVD conditions and risk factors also reported it to be low. For example, a cross-sectional study in Uganda among a mostly urban population found that nearly 75% did not know any risk factors and warning signs for stroke [12], and in another in rural and urban Mukono district, participants rarely mentioned smoking as a stroke risk factor [11]. In Cameroon, although moderate to high knowledge levels on CVD risk factors were reported, overall knowledge scores were equally low and gaps in warning signs for heart attack and stroke were found [20]. Moreover, studies have shown that even with the high prevalence of CVD risk factors such as hypertension in many SSA countries, awareness and control remains low [6, 21, 22] amidst low population knowledge [13, 23] affecting health care seeking, diagnosis and treatment. It is therefore imperative that interventions to improve population knowledge about CVD and its risk factors such as those premised on health promotion and awareness raising are implemented to contribute to prevention and control efforts.

In the study, major sources of advice on CVD risk factors among community members was in the order of health workers, friends or relatives, and radio or television similar to previous research [9, 12]. The fact that informal sources such as friends and relatives were a major source of information to community members could partly explain the low knowledge levels observed in this study and highlights the need for interventions to target such key influencers. Moreover, we found gaps in understanding of key recommendations for CVD preventive practices. For example, although community members knew that physical activity is important for CVD prevention, most did not know the recommended duration for this activity. Whereas over 90% of the population in this community as well as the country are reported to meet the World Health Organization physical activity guidelines, this is majorly through work related activities [5, 24, 25]. Education interventions should therefore go beyond increasing awareness on CVD risk factors but also inculcate an understanding of specific recommendations so that populations have comprehensive information to make informed lifestyle changes. This will maximize the public health benefits of recommended health practices as most are dependent on meeting the minimum thresholds.

Health workers and mass media channels should also play important roles to equip and empower communities with CVD prevention knowledge contributing to improved lifestyle practices. Over 80% of our respondent households had a mobile phone whose ownership was associated with being knowledgeable thus increasing the potential for mobile health interventions, which have shown promise for CVD prevention in low- and middle- income countries [26, 27]. Also, although community health workers were a less reported avenue for advice, our previous research has shown that they are acceptable [28] and thus could be a key resource to target to improve community awareness on CVD especially in low income settings through their routine health promotion activities. Indeed, systematic reviews have demonstrated that community health workers could be effective in tackling the burden of CVD in both low- and- middle income countries [29]. The effectiveness of community health workers could be attributed to their wider reach in many areas, rapport with community members, and delivery of information in the local language [29, 30]. Thus, the SPICES project is implementing interventions targeting community health workers [14] to improve community knowledge and CVD prevention practices [25] in Mukono and Buikwe districts.

A SSA systematic review found that knowledge on CVD and risk factors was significantly influenced by population studied, place of residence and exposure to health information [19]. Indeed, in this study, CVD prevention knowledge was significantly higher among adults with post-primary education, those involved in formal occupations, those from a high socio-economic index, and respondents who had ever received advice on healthy lifestyles. Higher education as a predictor of high knowledge on CVD has been reported in previous studies in SSA [9, 12, 19, 20] and in high income countries [31, 32]. Education levels are also associated with occupation with higher educated individuals involved in more formal occupations and more likely to belong to a higher socio-economic index. A study in Cameroon found that beyond high level of education, a high monthly income was associated with moderate-to-good knowledge on CVD [20]. Other studies have also identified urban residence as influential in acquisition of CVD knowledge attributed to access to health information and higher services proximity [33, 34]. Moreover, in our study, those who had received health information or advice had higher CVD knowledge. It thus remains paramount to fully equip communities with key and comprehensive information on CVD prevention as a key measure to deal with the disease.

### Study limitations and strengths

This study was limited by the lack of a validated questionnaire on CVD prevention knowledge and although there was a possibility of information bias, this was unlikely since questions asked were very specific to which respondents indicated agreement or not. On the other hand, we delved into assessing comprehensive knowledge reducing information and social desirability biases as opposed to most previous studies. Also, previous studies majorly looked at knowledge on individual CVD risk factors or conditions unlike our study which had a broader scope. The study had a large sample size of adults selected rigorously from a well-defined sampling frame and was conducted in an area similar in context and characteristics with other parts of Uganda increasing potential for generalizability. The results may also be generalizable to similar contexts in African countries at same level of development.

## Conclusion

This study shows very low CVD knowledge with major gaps around recommended physical activity duration, diet and whether hypertension is asymptomatic. Indeed, less than a fifth of study respondents were knowledgeable on CVD prevention which was significantly higher among those who had post-primary education, were in formal occupation and belonged to a high socio-economic index. In addition, respondents from households that had a mobile phone and those who had ever received advice on healthy lifestyles were significantly more knowledgeable about CVD prevention. Observed knowledge gaps should inform suitable interventions and strategies to equip and empower communities with sufficient information for CVD prevention.

## Supplementary information

**Additional file 1.** Household Demographics Questionnaire

**Additional file 2.** Spices Project Participant's Questionnaire

**Additional file 3.** STROBE Checklist

## Data Availability

The data used during the current study are available from the corresponding author on reasonable request.

## Abbreviations

APR: Adjusted Prevalence Ratio
CVD: Cardiovascular disease
SPICES: Scaling up Packages of Interventions for Cardiovascular disease prevention in selected sites in Europe and Sub-Saharan Africa;
SSA: Sub-Saharan Africa
