# Supplementary material for: Cardiovascular disease prevention knowledge and associated factors among adults in Mukono and Buikwe districts in Uganda
Source: BMC Public Health. 2020 Jul 22;20:1151. doi: 10.1186/s12889-020-09264-6 (PMC7374818; doi:10.1186/s12889-020-09264-6)
Supplement: Supplementary file 1 — Additional file 1. Household Demographics Questionnaire [file 12889_2020_9264_MOESM1_ESM.docx]

*SPICES Project Household Demographics Questionnaire*

*Page 1 of 4*

**Household Demographics questionnaire**

Record ID SPICES Project


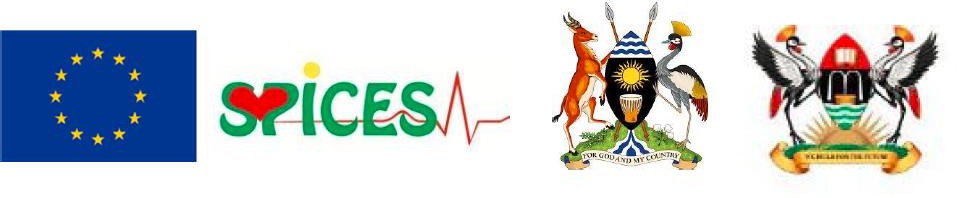


Household ID

Name of Research Assistant Time of starting interview H1: Name of the HH head

H2: Sex of Household head Male


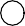

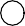


Female

H3: How old is the head of the household?

(complete years) (Don't know/declined....98)

H4: Is current respondent the household head? Yes No


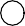

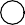


H5: If no, What is the respondent's relationship with Wife/Husband the Household head? Son/Daughter


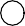

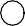

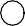

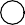

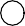

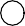

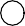

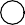

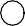


Son/Daughter-in-law Grandchild

Parent Parent in law

Brother/Sister Other (specify....) No response

Others, Specify..... H6: What is the main source of drinking water for Piped water


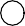

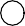

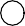

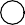

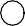

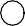

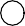

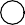

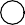

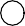

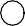

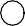


members of the household? Borehole

Protected dug well Unprotected dug well Water from spring Rainwater

Tanker water

Cart with small tank

Surface water (river/dam/lake/pond/stream) Bottled water

Other (specify.......) Don't know/declined

Others(specify...)

*Page 2 of 4*

H7: What kind of toilet facility do members of the Flush toilet


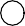

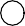

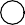

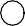

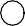

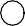

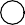

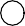

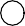

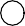


household usually use? Ventilated pit latrine

Pit latrine with slab

Pit latrine without slab/open pit Compositing toilet

Bucket toilet

Hanging toilet/ hanging latrine No facility/bush/field

Other (specify) Don't know/declined

H8: IF "FLUSH" OR "POUR FLUSH", PROBE:

Where does it flush to?

Others, Specify...... H8: Is the facility shared with other households? Shared


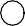

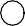

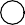


Not Shared

Don't know/declined

H9: Does the household have? Paraffin lamp

(Read to the respondent) Electricity

Mobile telephone

Non-mobile telephone (landline) Radio

Refrigerator Television

H10: What type of fuel does the household mainly use Electricity


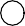

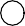

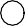

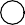

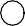

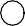

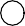

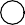

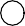

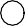

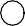

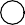


for cooking? Liquefied petroleum gas (LPG)

Biogas Kerosene Charcoal Wood

Straw/shrub/grass. Animal dung

No food cooked in household Other (specify)

Don't know/declined Coal lignite

Others specify.... H11: Record the main material of the dwelling floor Earth/sand


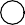

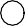

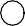

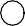

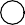

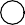

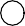

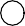

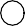

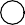

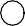


Dung

Wood planks Palm/bamboo Bricks

Parquet /polished wood Ceramic tiles

Cement Carpet

Other (specify)......

Don't know/declined ((Observe or ask))

Others specify....

Other specify...

Thatch/palm leaf Rustic mat Palm/bamboo/grass Wood planks Cardboard

Iron sheets Wood


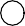

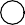

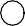

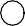

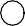

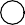

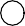

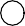

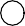

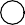

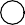

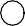

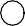


Calamine/cement fiber Ceramic tiles

Cement

Roofing shingles Other (specify)....

Don't know/declined ((Observe or ask))

H13: Record the main material of the walls. No walls Cane/palm/trunks Mud


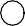

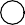

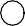

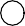

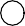

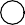

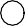

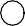

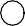

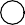

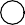

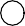

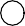

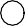

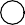

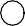


Bamboo/tree trunks with mud Stone with mud

Plywood Cardboard Reused wood Cement

Stone with lime cement. Burnt bricks

Unburnt bricks Cement blocks Wood planks Other (specify)...

Don't know/declined (Observe or ask))

Other specify....

H14: How many members reside in this HH? H15: How many rooms are there in the household?

(Don't know/declined..........98)

H16: How many rooms in the household are used for sleeping? (Don't know/declined..........98)

H17: Does any member of the household own:

Yes No DK/DTA A.OWatch
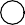

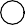

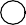
 B.OBicycle
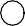

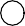

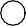


C.OMotorcycle or Scooter
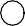

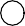

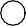
 D.OOxcart E.OCar or Truck

H18: Does any member of this household own any Yes

agricultural land? No

DK/DTA

other farm animals or poultry? No

DK/DTA

H20: Does any member of this household have a bank Yes account? No

DK/DTA

H21: How many members of the household are aged 25-70 years?

H21: What is your average household monthly income?

(Do not know.....98)

End time

Thanks you! ......End.
