# Supplementary material for: Cardiovascular disease prevention knowledge and associated factors among adults in Mukono and Buikwe districts in Uganda
Source: BMC Public Health. 2020 Jul 22;20:1151. doi: 10.1186/s12889-020-09264-6 (PMC7374818; doi:10.1186/s12889-020-09264-6)
Supplement: Supplementary file 2 — Additional file 2. Spices Project Participant's Questionnaire [file 12889_2020_9264_MOESM2_ESM.docx]

*SPICES Project Participant's Questionnaire*

**Spices Project Participant's Questionnaire**

Record ID SPICES Project


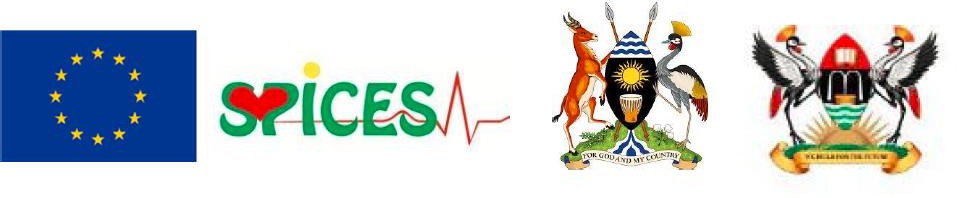


Name of Research Assistant Time Started interview Household ID

LD01: District Mukono


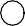

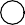


Buikwe

# Socio-demographic Characteristics

SD01 Participant’s ID SD02: Name of participant

(HH ID/0....)

(GIVEN NAME, SURNAME (ALL CAPS))

SD03: Is participant household head? Yes No


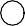

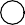


SD04: If no, what is the participant's relationship Wife/Husband


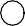

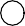

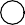

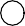

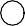

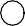

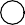

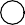

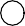


to household head? Son/Daughter

Son/Daughter-in-law Grandchild

Parent Parent in law

Brother/Sister Other (specify)... No response

Others, Specify...

SD05: Sex Male


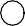

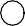


Female

SD06: How old are you now (age at last birthday)?

SD07: What is your current marital status? Never married Currently married Cohabiting Separated/Divorced Widowed


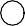

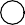

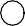

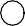

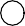


SD08: What is the highest level of education that you Never attended have attained? Primary


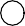

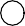

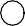

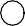

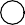

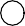


'O' Level 'A'-Level Tertiary University

SD09: What kind of work do you do? Farmer Merchant/Shop Student Housewife Health worker Fisherman Teacher


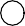

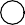

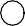

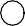

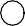

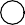

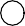

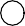

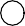


Other

Do not know/No response

Others, Specify.... SD10: What is your religion? Catholic


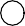

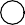

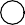

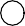

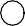

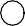

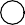

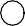


Protestant Pentecostal Muslim SDA

Other None

No response

Others, Specify...

SD11: How long have you been living (continuously) in this area? (IF LESS THAN 1 YEAR, ENTER "00")

SD12: Where have you lived for most of your adult Rural area


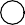

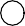


life (18+ years)? Urban/city

SD13: Where did you live for most of your childhood Rural area


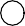

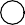


life (below 18)? Urban/city

# History of Raised Blood Pressure and Diabetes

HB02: Have you ever had your blood pressure measured? Yes


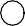

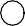

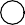


No

Don't know

HB03: Have you ever been told by a doctor or other Yes


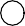

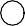


health worker that you have raised blood pressure or No hypertension?

# Knowledge:

**In this section, we would like you to say whether you agree with the statement read to you or not.**

KN01: Someone can have hypertension without having Yes any symptoms. No


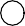

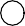

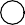


Don't know

KN02: High salt consumption can increase your risk of Yes developing hypertension. No


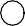

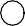

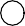


Don't know

KN03: Foods high in calories such as chips, sugary Yes


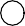

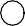

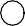


drinks, cakes are good for the heart. No

Don't know

KN04: Increased consumption of fruits and vegetables Yes increases your risk of cardiovascular disease. No


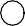

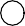

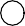


Don't know

KN05: Sitting for a long time puts you at risk for Yes


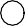

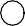

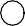


high blood pressure. No

Don't know

KN06: In order to reduce your risk of cardiovascular Yes


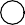

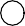

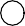


disease, moderate physical activity of at least 30 No

minutes is recommended every week. Don't know

HB06: Have you received any advice on any of the following?

Yes No

1. Salt reduction
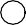

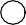

2. weight loss
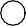

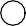

3. smoking cessation
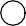

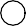

4. physical activity
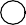

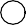

5. Alcohol reduction
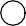

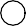


HB07: Who provided you with any of this advice? Community Health Worker / VHT

Health worker Friend / relative Other (specify) radio / television

Others (specify).....................................
